# Notes and News

**Published:** 1894-02-10

**Authors:** 


### NOTES AND NEWS.

Collected and Communicated.

The Mayor of Leeds presided at the fourth annual meeting of the District Council for the National Registration of Plumbers, at which a resolution was passed in favour of the Plumbers' Registration Bill now before Parliament in the interest of the public health. Mr. W. H. Bishop, the late Master of the Plumbers' Company, attended to represent the Court of the Company.

The Birmingham Press is taking up the question of out-patients raised in "Forward," the organ of the Birmingham Hospital Saturday Fund. The *Birmingham Post* justly points out that if the Queen's and General Hospitals of that city would agree to act from one standpoint with the co-operation of the Hospital Saturday Fund, the satisfactory solution of the difficulty would be in a fair way to be settled. Throughout the provinces there appears a determination to find a wise solution of the problem, and if the matter is not lost sight of it is probable that the provinces will be setting an example the metropolis will be constrained to follow.

Death has deprived us of one of the most distinguished surgeons of our time. Dr. Billroth, the great surgical operator of Vienna, died in that city on Tuesday last in the midst of his labours. Professor Billroth's health for years past had been in a precarious condition owing to fatty degeneration of the heart, yet his end came as a painful surprise to those associated with him. A great and continuously occupied operator, he carried his interests far afield, and was one of the most strenuous promoters of efficiency in any department of medicine upon which his influence could be brought to bear. As a teacher his following was very large, and not confined to persons of his own nationality. Doctors Czerny, of Heidelberg, Wölfler, of Gratz, and Eiselberg, of Utrecht, have all been numbered amongst his assistants. For Englishmen he has an especial interest from the fact that he was one of the few foreign medical men who upheld Sir Morell Mackenzie in his treatment of the Emperor Frederick of Germany.

The Croydon County Council are having it impressed upon them that a fever hospital must be erected as soon as possible. There is good promise that this will be more satisfactorily managed than many like institutions, as arrangements are being made for more efficient control at the temporary hospital as a commencement. One of the so-called "hospital scandals," which in this case is absurdly improbable on the face of it, has found its way into the Press in regard to the hospital now receiving patients from Croydon. The "scandal" alleges that three children were kept in the hospital for six weeks without being provided with a change of linen. The matter is being investigated, though it would seem hardly necessary to offer a denial even to a credulous public.

Four important differences were made in the working of the Carnarvonshire and Anglesey Infirmary at the last meeting of the General Committee. The completion of the alterations is expected in April. Under the new regulations out-patients will not be attended by the house surgeon, whose salary has accordingly been reduced from £105 to £70. An objection arose on this head at the meeting, on the score that the lesser salary would not secure a man of standing, and that a constant change would occur in the occupation of the post. This objection was met with the explanation that a permanent doctor of standing was not desired, seeing that the visiting staff possess these qualifications, and a younger man to carry out their directions was obviously preferable. Besides the change in the house surgeon's salary, the matron's remunerations has been fixed at £45 instead of £60, the provision of uniform being left to the House Committee's decision.

We have received the prospectus of the third Indiana State Conference on Charities and Correction, which will hold its meetings from the 20th to the 22nd inst. We call attention to this conference because we are confident that if the managers of the various hospitals, charities, relief agencies, and reformatory organisations within each County throughout England were to hold a County Conference every year much good would result. In America these State conferences open with an address by the President, and the appointment of the necessary committees to revise the rules and prepare resolutions, proceedings which are followed by the formal opening of the various sections. On the first day, child-saving and charity organisations, including the problem of the poor in cities, will be dealt with at the Indiana Conference; on the second day, reformatory work, prisons and gaols, including manual training in public schools, will occupy the morning, whilst asylum discipline and the relation of outside authorities to asylums will be discussed in the afternoon. On Thursday the morning will be devoted to questions affecting the poor laws, including out-door relief and travelling mendicants; whilst the afternoon will be devoted to intemperance in relation to crime and pauperism, the cure of inebriety, and the public control of the liquor traffic. England is the home of the voluntary system, and it is only proper that Englishmen should frequently exchange ideas with each other on these vital social problems. County Conferences, such as we have suggested, would infuse new life into many movements, arouse and fix public attention to the work that was being done, and so tend to diminish abuses and increase efficiency.

